# Comparison of Sulfamethoxazole Removal Efficiency Using Polyethersulfone Ultrafiltration Membrane Modified by Various Methods

**DOI:** 10.3390/ma17246247

**Published:** 2024-12-20

**Authors:** Asunción María Hidalgo, María Dolores Murcia, María Gómez, M. Mar Collado-González, María Claudia Montiel, Marta Martínez

**Affiliations:** 1 Department of Chemical Engineering, Faculty of Chemistry, Campus of Espinardo, University of Murcia, 30100 Murcia, Spain; md.murcia@um.es (M.D.M.); maria.gomez@um.es (M.G.); cmontiel@um.es (M.C.M.); marta.martinezc@um.es (M.M.); 2 Department of Cell Biology and Histology, Faculty of Biology, Campus of Espinardo, University of Murcia, 30100 Murcia, Spain; mdmcg1@um.es

**Keywords:** membrane modification, ultrafiltration membrane, chitosan, reduced graphene oxide, saline solutions, sulfamethoxazole, fouling

## Abstract

Nowadays, there is a growing interest in membrane modification processes to improve their characteristics and the effectiveness of their treatments and reduce the possible fouling. In this sense, in this work, a modification of an ultrafiltration membrane with three different materials has been carried out: reduced graphene oxide (rGO), chitosan and MgCl_2_. For both the native and the modified membranes, a study has been carried out to remove the emerging contaminant sulfamethoxazole (SMX). SEM and SEM-EDX analyses have been performed to confirm membrane surface modifications. In the characterisation of the membranes, it is noteworthy that the values of the permeability coefficient, A_w_, have been lower in the modified membranes, which is unexpected. Regarding the pollutant removal tests, the influence of pressure and initial concentration on permeate flux and rejections has been studied. Native membrane shows the highest permeate flux values. Comparing the modified membranes, the highest rejection values are obtained with the rGO-modified membrane, which can be explained by its greater hydrophilic character. Finally, a fouling study was carried out, verifying that in almost all cases, fouling occurs after the passage of the pollutant due to the blockage of the membrane pores.

## 1. Introduction

Polymeric membranes are versatile materials [1,2,3,4] that possess a wide variety of applications in fields such as the separation of chemical products, water purification and energy production. They are composed of synthetic or natural polymers whose porous structure and size distribution allow the separation of different compounds in a mixture depending on their size via the ultrafiltration (UF) process [5]. The chemical properties of the membrane can be exploited to enhance the separation process [6]. Despite the progress that exists in the membrane industry, there are some issues that still need to be addressed when it comes to large-scale applications.

Ultrafiltration is affected by both concentration polarisation and membrane fouling [7,8]. The latter can be considered the main factor that limits the use of membranes since it consists of the accumulation of various solutes on their surface or in their interior structure, which prevents the passage of the solution through it. Therefore, pores become blocked, and the operation flux decreases. In the specific case of ultrafiltration (UF) membranes, which mostly operate by crossflow filtration, membrane fouling causes organic compounds in the feed water to condense on the membrane surface and promote bacterial growth [9].

**Figure 1:**
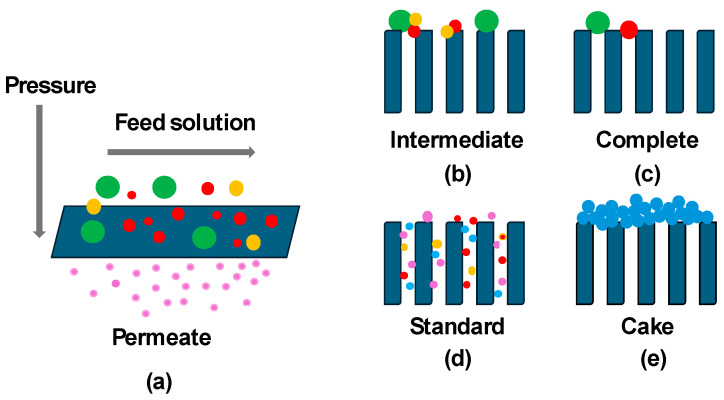
(**a**) Schematic mass transport mechanism of the UF membranes and types of fouling mechanism (**b**) intermediate pore blocking, (**c**) complete pore blocking, (**d**) standard pore constriction and (**e**) cake layer formation.

Different types of fouling can occur, such as fouling due to incrustations of salts, organic matter, colloidal mater, and microbial type. Fouling can accumulate as an additional membrane barrier or can cause an increase in the membrane wettability. Several UF tests have been carried out with polysulfone membranes that varied in molecular weight cut-off size (MWCO) to differentiate the fouling mechanisms. Figure 1 shows a schematic representation of the fouling mechanism during the ultrafiltration process. Both the characteristics of the membranes used and the feed solution influenced the fouling mechanisms [10]. Similar results were reported by Saeky and colleagues [9], who evaluated the biofouling behaviour in different MWCO UF membranes.

One of the approaches to reduce the fouling of polymeric membranes is to avoid the adsorption or adhesion of compounds on the membrane surface. This goal could be achieved by pretreatments, modifications of the membrane surface, chemical or physical cleaning and so on. Paying special attention to the modification of the membrane surface, in recent years, techniques have been developed to increase the hydrophilicity of the membrane with a mixture of compounds, grafting, surface chemical reaction or the incorporation of nanoparticles [11,12,13,14]. The dispersion of inorganic nanoparticles in the polymer matrix has shown great utility in improving the performance of nanofiltration and ultrafiltration membranes. Furthermore, the fact of being able to increase the hydrophilic groups of the membrane by incorporating a hydrophilic functional group on its surface can reduce fouling since the interactions of most of the foulants with the membranes are hydrophobic, so unwanted adhesion and adsorption interactions are reduced, and the free surface energy of the membrane is improved [15].

On the other hand, the performance of the membrane is strongly influenced by its chemical composition and mechanical resistance since these qualities guide the behaviour of the values of permeate, rejection and fouling fluxes. Surface charge, operating pH and pore structure also influence fouling behaviour. Furthermore, the identification of the mentioned parameters can be useful in predicting the relationship between the manufacturing mode of the membrane, the structure to be manufactured, the surface properties of the membrane and the performance that can be achieved [16]. This is why the study of various types of modification of the membrane surface can clarify the field of research developed so far and open new paths in the search for improving the characteristics of polymeric membranes for their use in large-scale applications.

Materials such as graphene and its derivatives have been used to reinforce these composite materials since they have better thermal, electrical and mechanical properties than other materials, and they are the most rigid and resistant material known [17]. Graphene oxide (GO) can increase the compatibility with organic polymers because it has several functional groups, such as carboxylic acids and hydroxyl groups, among others, which migrate to the surface of the polymeric membrane during the manufacturing process, thus improving the surface properties and hydrophilicity of the nanocomposite membrane [18,19].

Chitosan is an aminopolysaccharide obtained from the deacetylation of chitin, widely distributed in nature [20]. Chitosan is a hydrophilic, biocompatible, biodegradable, non-toxic biopolymer that shows antibacterial and antioxidant properties. The latter justifies the application of chitosan in desalination processes of aqueous media. Moreover, chitosan is a polymer enriched in hydroxyl and amine groups, which enhances its interaction with anionic pollutants. On the basis of all their advantages, chitosan has been used in the development of modified polymeric membranes for water purification [21] since they can retain pollutants such as water-soluble organics and metal ions [22]. Such water purifications are known as polymer-enhanced ultrafiltration (PEUF) processes.

Extensive research has been conducted on the modification of membranes with chitosan to enhance their antimicrobial properties and improve their sustainability in resisting fouling [23,24,25,26,27].

The addition of chitosan to polymeric membranes for their modification has also been studied by other authors, such as Darwish et al. [28], who modified polyacrylonitrile (PAN) and polyvinylidene fluoride (PVDF) ultrafiltration membranes. These researchers verified the performance of the modified membranes through filtration tests of aqueous solutions of humic acid (HA), observing an improvement in their hydrophilicity due to the deposition of chitosan particles within the pores of the membranes.

The addition of chitosan not only improves water purification but also improves the addition of nanoparticles to chitosan-enriched membranes. This is the case of the addition of graphene oxide (GO), ZnO, Ag, Cu and TiO_2_ to the chitosan polymer membranes that show an improvement in membrane adsorption, fouling performance, thermomechanical properties and contaminant retention.

Zhang et al. [29] investigated the modification of a polyethersulfone (PES) membrane (58 kDa) using water-soluble “metal–organic framework” (MOF) nanoparticles. The authors concluded that the size of the MOF nanoparticles did not affect the microporous structure of the separation layer, and the permeability of the modified membrane was significantly improved while maintaining high rejection.

Other authors, based on the procedure described in Zhou et al. 2014 [30], investigated the modified surface of polysulfone membranes by applying several treatments with salt solutions such as magnesium chloride [31].

In recent years, researchers have shown that the modification of polymeric membranes can be a powerful tool in the separation or elimination of water contaminants, such as heavy metals, dyes, some drugs, and other toxic compounds. This is a promising and novel approach in the field of water purification.

Membranes have been widely used lately for the removal of emerging pollutants from water. Among them, pharmaceutically active compounds (PhACs) are of particular interest due to their extensive use and the fact that traditional treatments cannot remove them efficiently. For instance, a previous work [32] investigated the removal of three pharmaceutically active compounds (atenolol, sulfamethoxazole and rosuvastatin) from water testing both nanofiltration and ultrafiltration membranes.

The aim of this study has been to carry out a comparison of three different modification techniques for a polymeric ultrafiltration membrane using three different types of modification agents: one of chemical type (a saline solution of magnesium chloride), another of biochemical type (chitosan solution) and another of physical type, a suspension of reduced graphene oxide. The modified membranes have been characterised, and their efficiency in the separation process of an emerging pollutant such as sulfamethoxazole (SMX) has been evaluated.

## 2. Materials and Methods

### 2.1. Materials

Sulfamethoxazole (SMX) (C_10_H_11_N_3_O_3_S, M_w_ 253.28 g/mol, ≤100% purity) was purchased from Sigma-Aldrich (Barcelona, Spain). Magnesium chloride hexahydrate (MgCl_2_∙6H_2_O, M_w_ 203.3 g/mol) was purchased from Panreac Quimica S.A.U. (Barcelona, Spain), and reduced graphene oxide was kindly provided by the Department of Chemical and Environmental Engineering from the University of Cartagena. Chitosan (M_w_ 2 kDa, 98.2% DDA) was kindly provided by Chibio (Qingdao, China). The rest of the reagents used for the development of this work were of analytical quality grade: MilliQ water (Merck, Wicklow, Ireland), acetic acid and sodium hydroxide (NaOH).

For the performance of the current experimental work, Alfa Laval (Madrid, Spain) provided ultrafiltration membranes GR95PP. The technical specifications of the membranes, provided by the manufacturer, are included in Table 1.

### 2.2. Methods

#### 2.2.1. Methods of Membrane Modification

**Figure 2:**
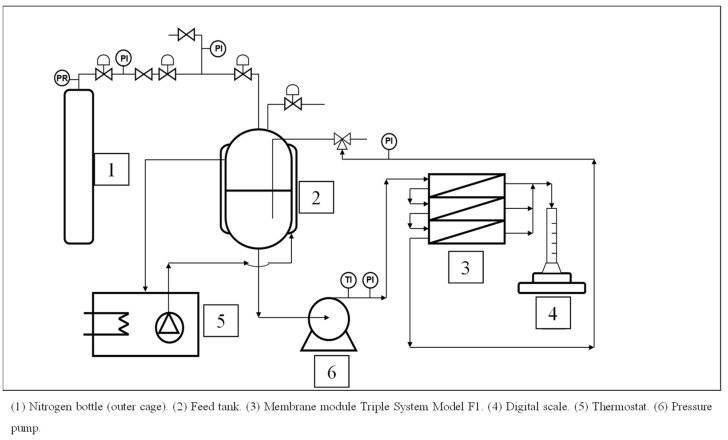
Diagram unit flow for the Triple System Model F1 membrane module. (PI) Pressure Indicator, (PR) Pressure Regulator and (TI) Temperature Indicator.

An experimental unit for ultrafiltration tests, the Triple System Model F1 membrane module from MMS (Urdorf, Zurich, Switzerland), was employed, enabling the collection of permeate flux samples at different manually regulated working pressures. Figure 2 shows a diagram of the experimental unit.

Three different methods were implemented for membrane modification using three different materials: MgCl_2_, rGO and chitosan. Each method is detailed below.

##### Modification of Membranes with Magnesium Chloride (MgCl_2_)

Initially, 800 mL of a MgCl_2_ solution (0.5 g/L) was placed in the module feed tank. Then, the MgCl_2_ solution was forced to pass through the membrane under a constant pressure of 8 bar. This process lasted for 3 h. A record of the mass flux rates was kept comparing the permeability with that of the native membranes.

After performing tests with SMX, which was used as the contaminant, and with distilled water, the second modification of the same membranes was carried out with a MgCl_2_ solution at 1 g/L. The membrane modification procedure was identical to that described above, but the filtration procedure lasted 2 h. Likewise, the mass flux rates obtained during the process were recorded.

##### Modification of Membranes with Reduced Graphene Oxide

As a preliminary step, it was necessary to enhance the adhesion capabilities of graphene. To do so, a mixture of reduced graphene and water (distilled) was sonicated (equipped with a flat-tipped probe of 1.27 cm in diameter at an amplitude of 30% in pulses of 15 s ON and 15 s OFF for approximately 10 min) by using an ultrasounds device, Branson 450 D sonicator, Emerson Ultrasonic Corporation (Dansbury, CT, USA). Next, the membrane was positioned in the Buchner funnel, with the active side facing upward. Vacuum filtration was performed by pouring the rGO mixture to cover the entire surface of the membrane. Subsequently, the resulting mixture was transferred to a container, and the membranes were immersed until completely covered. Counterweights were used to prevent the membranes from bending and being uncovered by the solution. Three different native GR95PP membranes were employed for these modification tests, distinct from those used in previous experiments.

##### Modification of Membranes with Chitosan

Chitosan was solved in 0.3% acetic acid in MilliQ water (103 mL). Chitosan was allowed to be dissolved for 12 h before adding 100 mL of MilliQ water to the solution. The final chitosan concentration was 3.98 mg/mL, and this solution was used to modify the native GR95PP membrane, as indicated below.

The modification process consisted of the following under vacuum filtration process, all of which were carried out at 12 bar of pressure.

**Figure 3:**
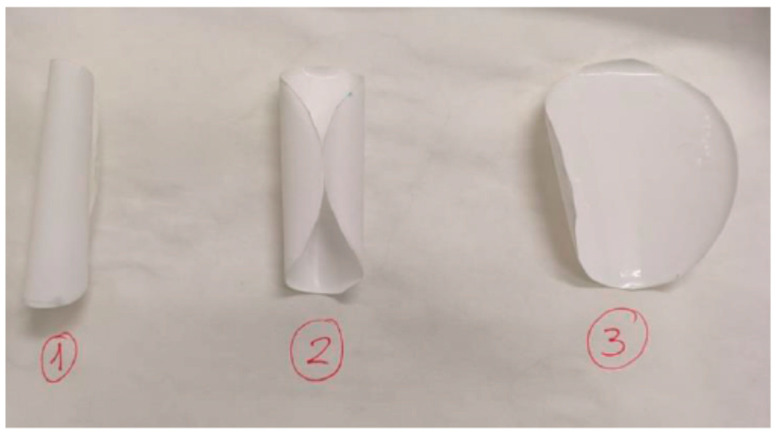
Chitosan-modified membranes before being introduced into the module.

Firstly, 20 mL of chitosan solution was forced to pass through the membrane. The membrane was then placed in an oven at 40 °C for 30 min. Then, 100 mL of NaOH 1 M in ethanol 50% was vacuum filtered through the membrane. Another 30 mL of the same solution was forced to be vacuum-filtered through the membrane. Finally, 30 mL of ethanol 50% was vacuum-filtered through the membrane to remove the excess NaOH from the membrane’s active surface. Three native membranes were modified following this procedure. Figure 3 shows the appearance of the membranes after the chitosan modification processes.

Once the filtrations were completed, the membranes were allowed to dry at 25 °C for 10 min. Then, the membrane was placed in the membrane module to perform the filtration assays, as explained below.

#### 2.2.2. Scanning Electron Microscopy

The ultrastructure of the membranes, either modified or native, was analysed by scanning electron microscopy (SEM). To do so, the S-3500 N scanning electron microscope (Hitachi) was used. The analysis was performed at the Polytechnic University of Cartagena (UPCT). A variable pressure SEM scanning electron microscope, model HITACHI S-3500N (Hitachi High-Technologies Corporation, Tokyo, Japan), was used. This machine has a resolution of 3 nm (high vacuum mode) or 4.5 nm (low vacuum mode). Its pressure range can vary from 1 to 270 Pa, and it has a digital image resolution of up to 2560 × 1920 pixels [33]. It detects secondary electrons, variable pressure secondary electrons, and Robinson backscattered electrons. It is equipped with an EDX XFlash 5010 analysis system X-ray detector (Bruker AXS, Karlsruhe, Germany) [34].

#### 2.2.3. Filtration Experiments

##### Solvent Permeability Test

For the determination of membrane permeability, the membranes were immersed in a mixture of tap water and distilled water to activate their active surface. Then, distilled water was passed through the membranes at pressures ranging from 6 to 12 bar by using the experimental unit for ultrafiltration tests, Triple System Model F1 membrane module (MMS). The pressures used varied depending on the type of membrane modification. Pressures of 6, 8 and 10 bars were used for the native membrane and those modified with salts and chitosan. For the membrane modified with reduced graphene oxide, the pressures tested were 8, 10 and 12 bar. The tests typically spanned for 20 min on average for chitosan- MgCl_2_- and non-modified membranes and 30 min on average for graphene oxide-modified membranes since the permeate flux through the latter was lower, meaning that a larger time for data collection was needed. From each experiment, values were recorded for permeate weight, inlet temperature, and inlet and outlet pressures at various time intervals (2, 3 or 5 min) depending on the pressure used for the test and the membrane permeability.

##### Tests with Sulfamethoxazole Pollutant

SMX solutions (800 mL) at concentrations equal to 12.5, 25 and 50 ppm were used. The duration of the tests was approximately 20 min for MgCl_2_-, chitosan- and non-modified membranes. Nevertheless, in the case of graphene oxide-modified membranes, the test lasted for 70 min. The pressure range tested varied between 6 and 10 bar. In the same way, the pressure increased to 12 bar in the case of graphene oxide-modified membranes. Permeate samples were collected at predetermined intervals for each test. The monitoring consisted of recording permeated weight, inlet temperature, inlet and outlet pressure values, and measured absorbance values for each sample taken. Once a steady state was reached, the test was considered finished. At the end of each test, the remaining volume of the aqueous solution in the tank, as well as its absorbance at 275 nm, was measured using a UV–Vis spectrophotometer (Evolution 300, Thermo Electron, Thermo Fisher Scientific (Waltham, MA, USA)). The absorbance at 275 nm was used to calculate the SMX concentration in the feed, permeate and reject samples. The calibration curve for SMX was C = 15.529 × Abs + 0.9445; R^2^ = 0.9979.

As a cleaning procedure, distilled water was passed through the membrane to compare the flux and permeability values with those obtained in tests prior to the SMX passage.

#### 2.2.4. Data Processing

The data shown in the results correspond to the average and standard deviation of the raw data collected during the experiment. All the experiments were run in duplicate, and the maximum calculated standard deviation using the Regress Excel tool was 3.05%.

## 3. Results

### 3.1. Membrane Modification

**Figure 4:**
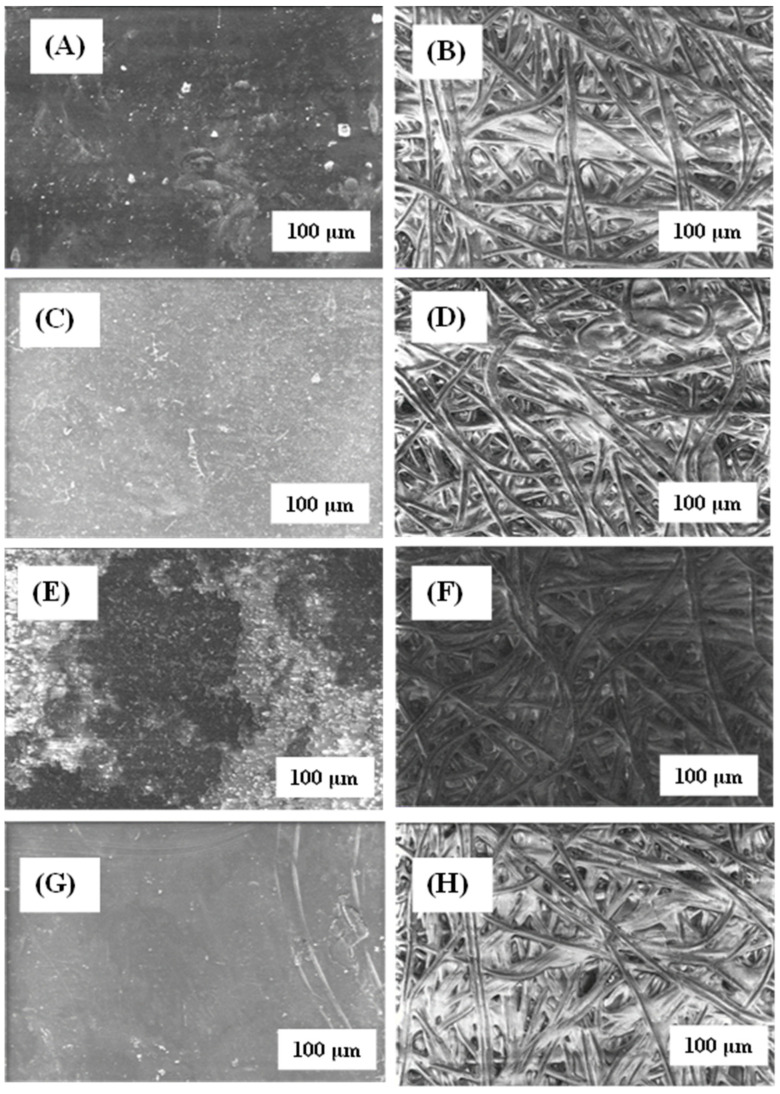
Comparison of SEM images of the GR95PP membrane. The micrographs (**A**,**C**,**E**,**G**) correspond to the active layer membrane and (**B**,**D**,**F**,**H**) to the non-woven layer. Micrographs (**A**,**B**) correspond to the native membrane and (**C**,**D**) to the MgCl_2_-modified membrane after filtering the SMX, (**E**,**F**) rGO-modified membrane after filtering the SMX and (**G**,**H**) chitosan-modified membranes after filtering the SMX.

**Figure 5:**
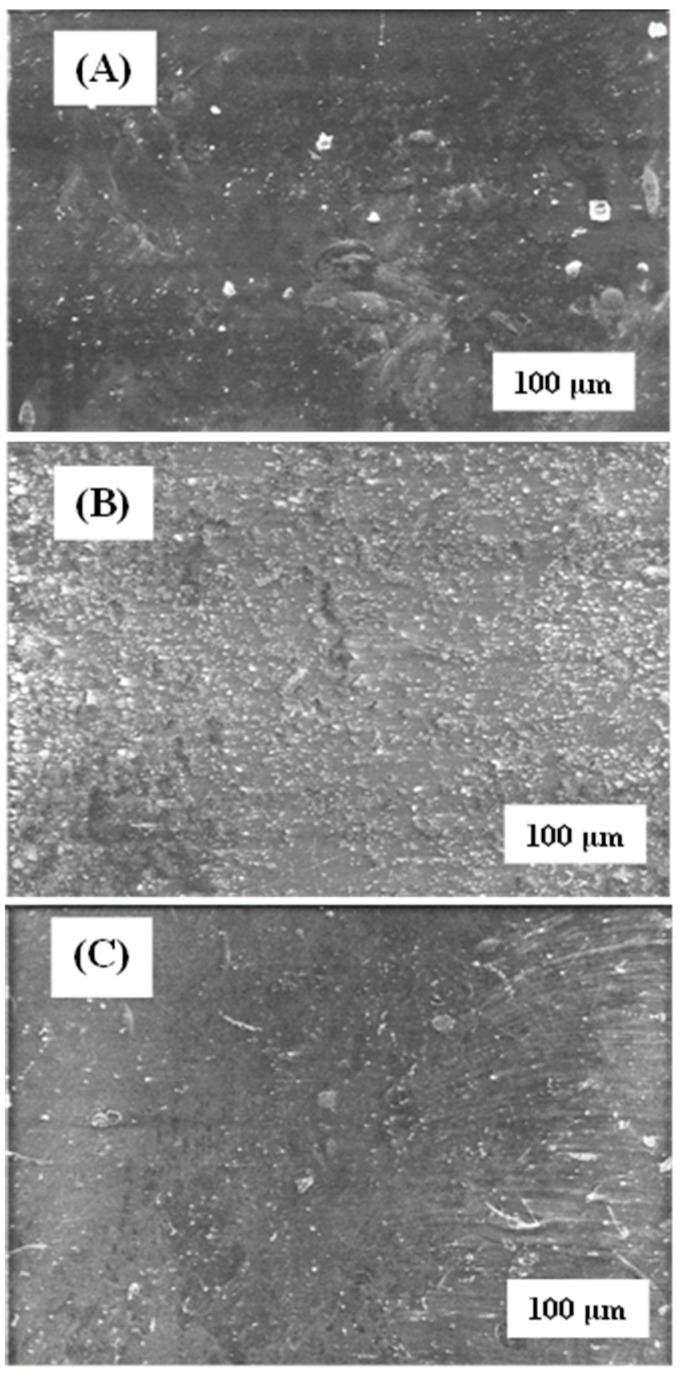
Comparison of SEM images of the GR95PP membrane. The micrographs correspond to the active layer membrane of (**A**) the native membrane, (**B**) the rGO-modified membrane and (**C**) the chitosan-modified membrane.

The microstructure of the active surface of native or modified membranes was analysed by SEM [35], either before or after filtering the SMX (Figure 4). Native membrane showed a coarse appearance that increased its roughness once it was modified with rGO (Figure 5B) and chitosan (Figure 5C). Such a roughness increment could be explained by the solute adherence on the membrane surface. Nevertheless, the appearance of the non-woven surface of the membranes (Figure 4B,D,F,H) was very similar regardless of the treatment applied, except in the case of rGO modification, which resulted in the membrane darkening (Figure 4F). Again, such a darkening process could be interpreted as the rGO adherence on the membrane.

Interestingly, SMX filtration produced different effects on the appearance of modified membranes. Reduced graphene oxide-modified membranes resulted in light and obscure patches on the surface of the active membranes, which may indicate that the passage of the aqueous SMX solution removed the rGO that did not adhere firmly to the surface (Figure 4E). It is to be noted that the appearance of the membrane after filtering the SMX became rougher, which could indicate the presence of fouling after the passage of the SMX. Similar results have been obtained by modifying membranes with rGO in the elimination of ibuprofen [36]. A completely opposite effect can be seen in the case of MgCl_2_ and chitosan-modified membranes, which became smoother after SMX filtration (Figure 4C,G).

**Figure 6:**
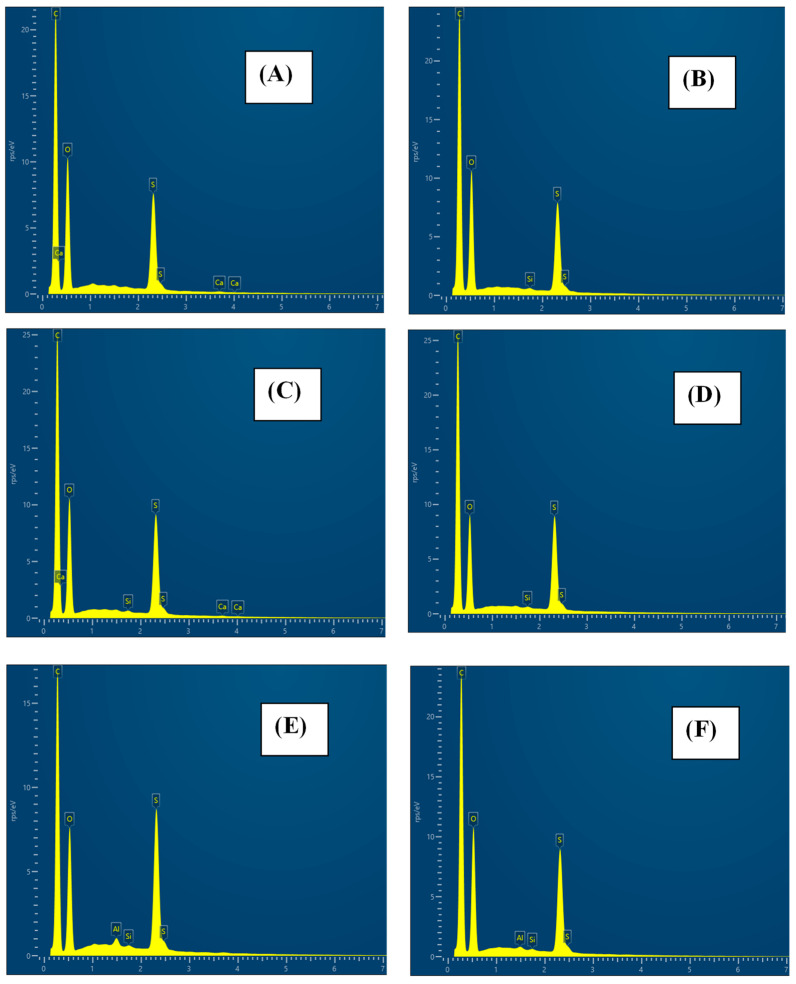
Comparison of SEM-EDX spectra for the native and the modified membranes before and after SMX filtration. (**A**) GR95PP native before, (**B**) GR95PP MgCl_2_ after, (**C**) GR95PP rGO modified before, (**D**) GR95PP rGO modified after, (**E**) GR95PP chitosan before, (**F**) GR95PP chitosan after.

Additionally, SEM-EDX analysis was performed on the native membrane before filtering the SMX, and the membranes were modified with rGO and chitosan either before or after filtering the SMX. Regarding MgCl_2_ membranes, due to technical limitations, SEM-EDX analysis was only possible for those membranes after having filtered the SMX. Figure 6 shows the SEM-EDX spectra of native and modified GR95PP membranes. The spectra obtained in such analysis were used to extract the abundance, in percentage by weight, of different elements (Table 2).

Comparing the composition of the membranes before filtration, it is observed that the C and S content increased in the modified membranes. This is the greatest increase in C in the rGO-modified membrane and the greatest increase in S in the membrane modified with chitosan. On the contrary, the O content decreased in all modified membranes, with the lowest value being that of the chitosan-modified membrane. Filtering SMX through the modified membranes changed their composition. In the case of the membrane modified with rGO, the C content increased, whereas the O content decreased, which could be interpreted as the retention of the pollutant since the percentage of C in the SMX represents 35.7% of the molecule composition, while O represents 10.7%. Nevertheless, the unchanged value of S abundance in the membrane after filtering the SMX seems to indicate that any S-rich compound is getting retained in the membrane, which is not the case of SMX since its S richness is 3.6%. In the case of the membrane modified with chitosan, the abundance of C and O increased after the SMX filtration, with the O element showing the greatest increase after the filtration process being 2.2 percentual units. Moreover, S abundance in the membrane showed a reduction in 2.3 percentual units after the filtration process. Regarding the composition of the MgCl_2_-modified membrane after the filtration process compared to the native one, whereas the C abundance increased (1.4%), the abundance of O and S showed a reduction in 0.9 and 0.5 percentual units, respectively.

### 3.2. Macroscopic Analysis Through Operational Parameters

The macroscopic characterisation of the membranes, native and modified ones, has been performed through operational parameters. Distilled water was used before and after filtering SMX.

#### 3.2.1. Permeability Test

Permeate flux (PWF) is an important parameter in the design and economic feasibility analysis of the membrane separation process. The study of the membrane permeability (native and modified) before and after the filtration process represents crucial data. The variation of the permeate flux with the pressure can be partly explained by the solution-diffusion model [37].

**Figure 7:**
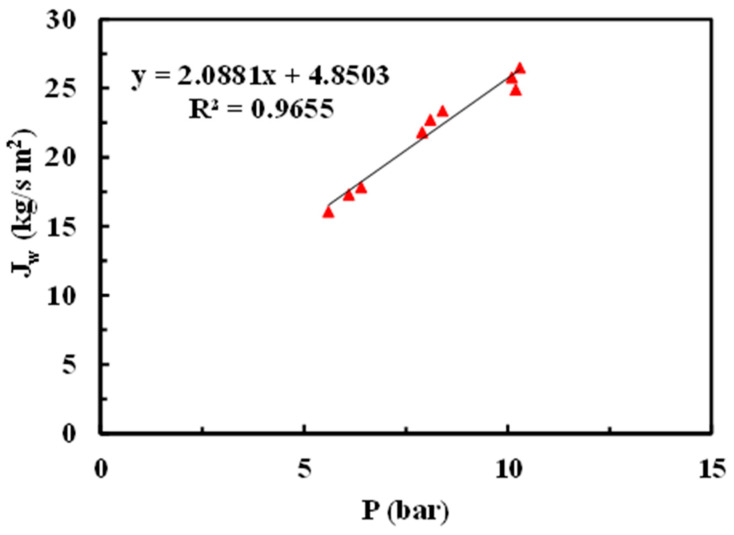
Permeability test of pure water (native membrane).

Figure 7 represents the permeate mass flux variation with operational pressure from the native membrane.

Table 3 shows a comparative study of permeate flux values (L/m^2^h) for GR95PP native membrane.

Permeability is a measure of the flux through a membrane. Equation (1) shows the relationship between the flux of the solvent permeate (in this case, distilled water) and the pressure gradient, which is the driving force across the membrane.

J_w_ = A_w_ ∙ (ΔP − ΔΠ) (1)

where J_w_ is the permeate water mass flux (kg/m^2^∙s), A_w_ is the membrane permeability to water (s/m), ΔP is the hydraulic pressure gradient across the membrane (Pa), and ΔΠ is the osmotic pressure gradient across the membrane (Pa). It is possible to disregard ΔΠ compared to ΔP since the tests have been conducted with distilled water, and no salts or organic solutes have been used. Therefore, Equation (1) can be written as Equation (2).

J_w_ = A_w_ ∙ (ΔP) (2)

J_w_ is plotted against ΔP, and a least squares fit is performed to obtain A_w_, which is equivalent to the slopes of the lines obtained from the fit (Table 4). It is worth noting that the Aw of the native membrane was reduced after any of the applied modifications, which is unexpected, as these modifications were intended to increase the hydrophilicity of the native membrane [17,18]. The A_w_ values of the modified membranes decreased after being modified, following the order: rGO< MgCl_2_ < chitosan.

The observed unexpected trend can be explained as a result of the interactions between the hydrogen from the carboxyl and hydroxyl groups present in rGO and the oxygen of the sulfone groups present in the polyethersulfone membrane.

The values of the permeability coefficient of the membrane after the passage of the pollutant only increase for the membrane modified with magnesium chloride (1.0 g/L) salt solution. This will be further discussed in Section 3.3.

#### 3.2.2. Sulfamethoxazole Removal

In order to study the behaviour of the modified membranes compared to the native ones, two experimental series of tests have been conducted, investigating the influence of operating pressure and SMX concentration on permeate mass flux values (J_p_). Additionally, the selectivity of the membranes has been examined through their rejection coefficients.

To characterise the selectivity of the membranes, a rejection coefficient (R), defined as the percentage ratio between the variation in the SMX concentration between the feed solution and the permeate solution and the concentration of the feed solution, is calculated according to Equation (3). The rejection coefficient expresses the membrane’s ability to remove a particular solute.

*R* (%) = (*C_a_* − *C_p_*)/*C_a_* ∙ 100 (3)

where C_a_ is the concentration of the feed solution in mg/L and C_p_ is the concentration of the SMX in the permeate solution in mg/L.

**Figure 8:**
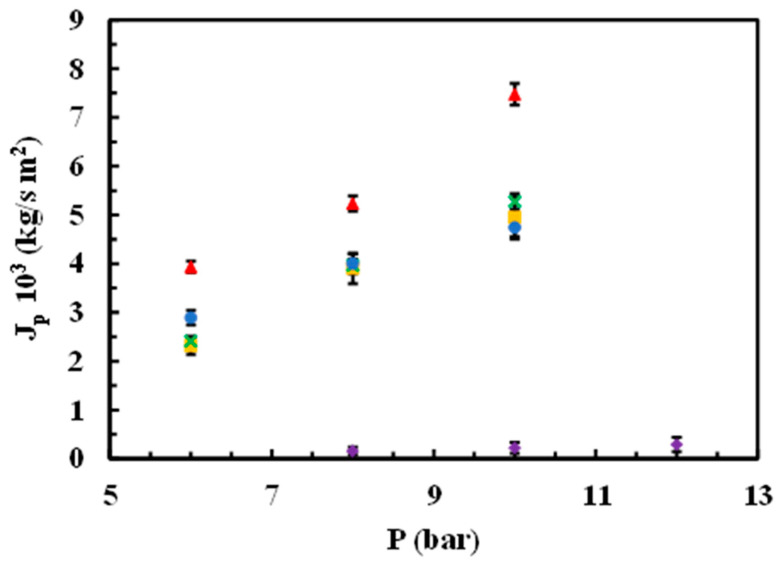
Influence of operating pressure on the permeate mass flux from a 25 ppm aqueous solution of the SMX for both native and modified membranes. (▲) Native, (●) modified chitosan, (■) modified MgCl_2_ 0.5 g/L (**x**) modified MgCl_2_ 1 g/L, (♦) modified rGO.

Figure 8 illustrates the influence of operating pressure on permeate mass flux using an SMX solution with a concentration of 25 ppm for both the native membrane and the modified membranes.

The effect of the operating pressure and SMX concentration on the permeate J_p_ values was analysed. Regarding the former, when using a solution of SMX at 25 ppm, the native membrane exhibited the highest permeate Jw up to 10 bar of pressure, while the lowest permeate J_p_ corresponds to the rGO-modified membrane up to 12 bar of pressure. For the sake of completeness, the permeate J_p_ for MgCl_2_- and chitosan-modified membranes was very similar between themselves, showing a positive correlation with the operational pressure in the range from 6 to 10 bar (Figure 8).

**Figure 9:**
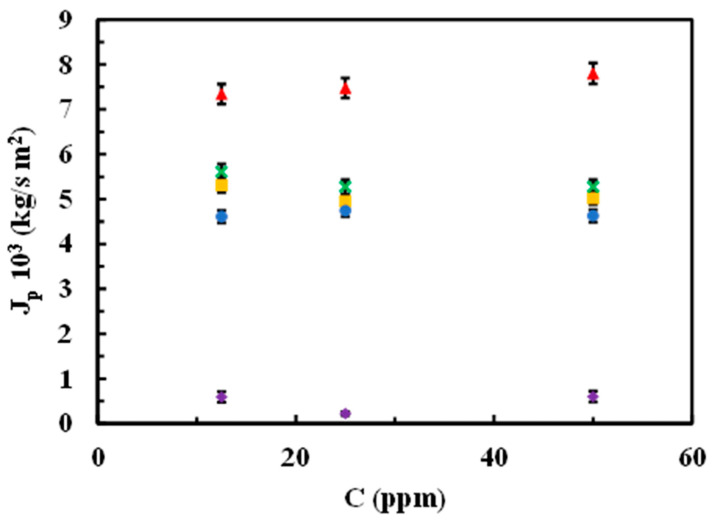
Influence of SMX concentration on permeate mass flux at 10 bar for both native and modified membranes. (▲) Native, (●) modified chitosan, (■) modified MgCl_2_ 0.5 g/L (**x**) modified MgCl_2_ 1 g/L, (♦) modified rGO.

Figure 9 depicts the relationships between permeate mass flux from aqueous SMX solutions and the three concentrations used for experimentation at an operating pressure of 10 bar for both the native membrane and the modified membranes.

Regarding the effect of the SMX concentration on the J_p_ at a fixed operational pressure (10 bar), again, the highest J_p_ value corresponded to the native membrane, and the lowest one corresponded to the rGO-modified membrane at any of the SMX concentrations in the range from 12.5 to 50 ppm. Modified membranes with MgCl_2_ and chitosan resulted in similar results that showed almost unchangeable values, regardless of the SMX concentration, in the same concentration range (Figure 9).

**Figure 10:**
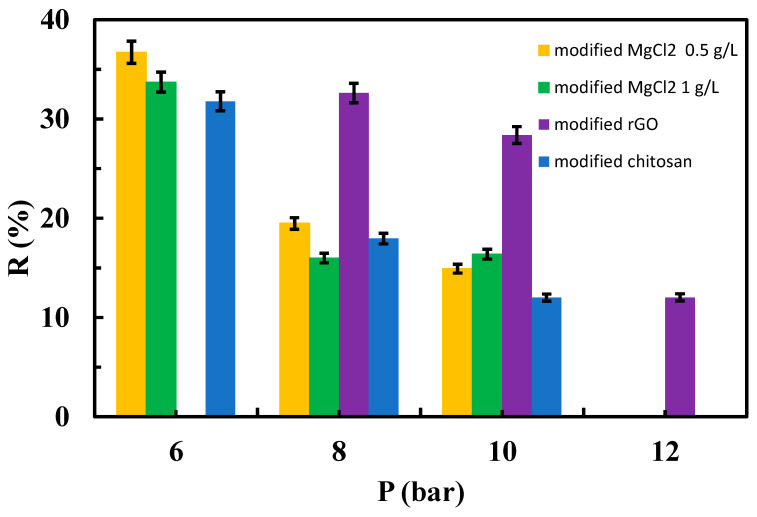
Influence of operating pressure on the rejection coefficient from a 50 ppm aqueous solution of the SMX for modified membranes.

**Figure 11:**
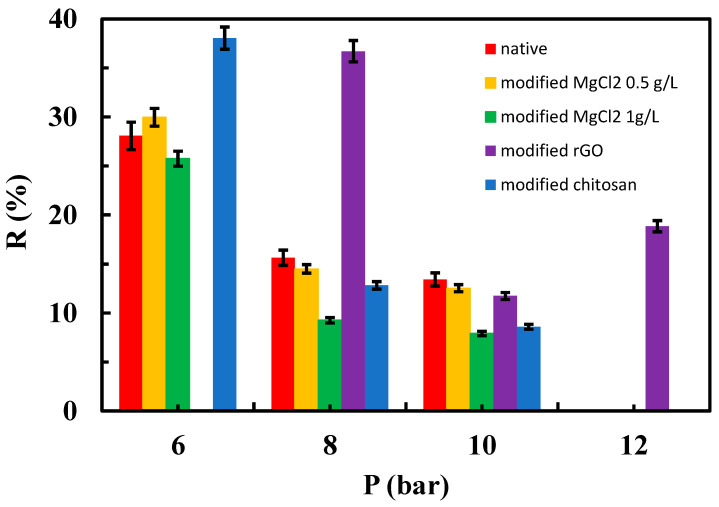
Influence of operating pressure on the rejection coefficient from a 25 ppm aqueous solution of the SMX for native and modified membranes.

**Figure 12:**
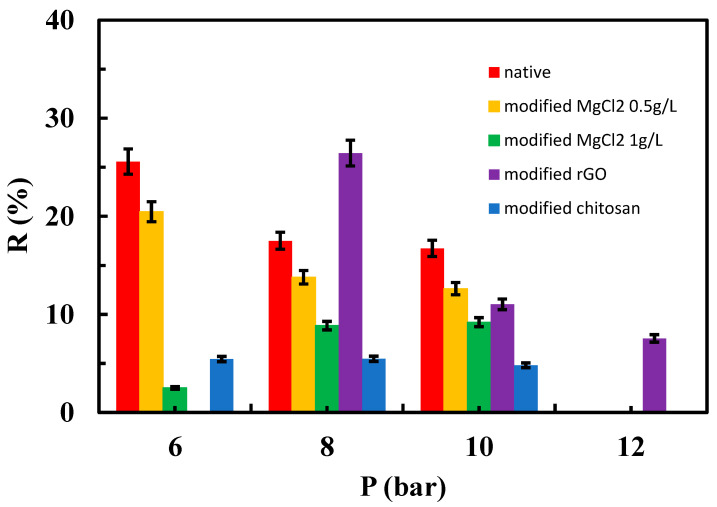
Influence of operating pressure on the rejection coefficient from a 12.5 ppm aqueous solution of the SMX for native and modified membranes.

Figure 10, Figure 11 and Figure 12 depict the relationships between the rejection percentages obtained using different aqueous SMX solutions with concentrations of 50, 25, and 12.5 ppm for both the native and modified membranes at various tested operating pressures.

The rejection of organic compounds is controlled by both the membrane and solute properties, where the major rejection mechanisms are size exclusion, electrostatic interactions and non-electrostatic interactions, which include hydrophobic interactions and the formation of hydrogen bonds [45].

Interestingly, rejection coefficient values were quite similar for native membranes regardless of SMX concentration. However, different results were obtained in the case of the modified membranes, especially when the system operated at 6 bar (Figure 10, Figure 11 and Figure 12). Note that at 25 ppm (concentration of feed tank/solution) of SMX, MgCl_2_ at 0.5 g/L, rGO, and chitosan-modified membranes showed higher rejection coefficient values than native membranes at 6 and 8 bar (Figure 10). In this case, the influence of MgCl_2_ concentration was noticeable in the membrane modified with this salt, being more effective the MgCl_2_ at 0.5 g/L, which indicates that varying the amount or concentration of the material used for membrane modification can be of interest to evaluate the performance, as previously suggested by other authors [29]. At the lowest concentration of the SMX assayed (12.5 ppm), a higher rejection coefficient value than that of the native membrane was only true for rGO-modified membranes when operating at 8 bar (Figure 12).

It can be stated that the rejection coefficient for the native membranes decreases as increasing the operational pressure in a non-linear way. In fact, rejection coefficients seem to show no change when operating at 8 and 10 bar, regardless of the SMX concentration (Figure 11 and Figure 12). Interestingly, membranes modified with MgCl_2_, both 1 and 0.5 g/L, show the same trend as the native membranes in the presence of 25 ppm of SMX (Figure 11), whereas at 12.5 ppm of SMX, membranes modified with 1 g/L MgCl_2_ showed its lower rejection coefficient when operating at 6 bar. On the contrary, the rejection coefficient for chitosan-modified membranes decreased with increasing pressure when in the presence of 25 ppm of SMX, whereas in the presence of 12.5 ppm of SMX, the rejection coefficients seem unchangeable regardless of the operating pressure (Figure 12). Unexpectedly, the rejection coefficient for rGO-modified membranes did not follow the trend described for the native membranes. In fact, in the presence of 25 ppm of SMX and operating at 12 bar, the rejection coefficient showed a sharp increment (Figure 11). Moreover, in the presence of 12.5 ppm of SMX, the rejection coefficient decreased as pressure increased more prominently as the native membrane did (Figure 12).

Previous studies have reported that the rejection of organic compounds is also controlled by solute hydrophobic interactions, where hydrophobic membranes show lower solute rejection due to the adsorption of the compounds onto the membrane surface and facilitate diffusion into the permeate side, leading to lower rejection [46,47,48]. There was a clear increase in SMX rejection with the increase in membrane hydrophilicity (due to the modification of the membrane with rGO) [36].

The obtained results could also be explained by the negative charge present in rGO-coated membranes, which, as a result of the negative charge of SMX, leads to an increase in the repulsion of modified membranes and SMX at neutral pH, obtaining higher rejection values.

### 3.3. Fouling Parameters

The A_w_ was calculated again for each membrane once the SMX was filtered. The comparison of the A_w_ before and after filtering the SMX through the membrane (Table 4) can be used to determine membrane fouling. Interestingly, A_w_ showed an increase of 57% in the case of the membrane modified with 1 g/L MgCl_2_, while the other modifications resulted in a decrease in A_w_, specifically by 36.8% in the case of modification with rGO and by 58.6% when the membrane was modified with chitosan.

These results are somewhat surprising as it is suggested that modification of the membrane with nanocompounds results in an increase in the flux through it [49,50]. It has been suggested that grafting carboxylic groups onto polymeric membranes led to an increase in the pore size and, consequently, in the permeate flux [51]. Modifications carried out with graphene and its derivatives, such as reduced graphene oxide, also resulted in an increase in permeate flux in the membranes [19,52]. In the case of the modification of membranes with chitosan, which increases the presence of hydrophilic groups in the membranes, a higher solute rejection percentage has also been reported [28]. Nevertheless, the discrepancy between the results obtained in the current work and what has been reported in the literature in relation to the membrane modification with rGO and chitosan seems to have its explanation in the difference between the membrane pore size and the size of the SMX molecule. The fact that the SMX is much smaller than the pore size can lead to the blockage of the membrane pores due to penetration of the SMX into them [53,54,55]. The latter successfully explains the decrease in the permeate flux after filtering the SMX, and it can be described as membrane fouling. On the other hand, the membrane modification with 1 g/L of MgCl_2_ may have caused either a decrease in fouling tendency or a pore opening due to filtration processes, as this membrane has been used three times as many times as the others.

According to other studies, these phenomena were not observed because the increased hydrophilicity of the membrane favoured the formation of hydration layers, which reduced the interactions of the SMX with the membrane surfaces [56,57].

## 4. Conclusions

The ultrafiltration membrane GR95PP has been successfully modified with rGO, chitosan and MgCl_2_. SEM and SEM-EDX analyses have confirmed the membrane modification, which is more noticeable in the case of rGO. Contrary to the expected results, the water permeability coefficients of the modified membranes were lower than those of the native membrane, with the membrane modified with rGO having the lowest A_w_ value. Interestingly, in the removal of the emerging SMX sulfamethoxazole, this modified membrane also showed higher SMX rejection coefficients than the native membrane in certain experimental conditions (low pollutant concentration and intermediate operating pressures).

When characterising the membranes (native and modified) by solvent permeability and comparing the values of the permeability coefficient obtained in the initial tests and after the passage of the pollutant through the membranes, an increase in permeate flux was observed in the membranes modified with magnesium chloride saline solution, while the membranes modified with rGO and chitosan showed a decrease in permeate flux, with those modified with chitosan showing the greatest decrease in flux, indicating a greater tendency to fouling with respect to the rest of the modifications.

To sum up, in order to obtain high permeate flux, the native membrane is more efficient, while the one modified with rGO can reach higher rejections in mild operating conditions. MgCl_2_-modified membrane seems to be a good choice to avoid membrane fouling, while the other modifications need improvement in this sense, and further research is required.

## Figures and Tables

**Table 1:** Technical characteristics of GR95PP membrane.

Characteristics	Technical Specifications
Supplier’s signature	Alfa Laval
Designation	GR95PP
Filtration type	Ultrafiltration
Molecular Weight Cut-Off (kDa)	2
Active layer	Polyethersulfone
Support material	polypropylene
Operating pressure (bar)	1–12
Maximum tolerable pressure (bar)	10
Tolerated pH range	1–13
Temperature range (°C)	5–75

**Table 2:** Weight percentage values of the spectra from SEM-EDX images.

	Membrane	C (%)	O (%)	S (%)	Ca (%)	Si (%)	Al (%)
	Native	67.1	21.9	10.9	0.2	-	-
Before	MgCl_2_-modified						
filtering	rGO-modified	68.8	19.7	11.3	0.2	0.1	-
SMX	Chitosan-modified	67.5	18.3	13.7	-	0.1	0.5
After	MgCl_2_-modified	68.5	21	10.4	-	0.1	-
filtering	rGO-modified	71.1	17.5	11.3	-	0.1	-
SMX	Chitosan-modified	67.9	20.5	11.4	-	0.1	0.2

**Table 3:** Permeate water flux obtained by different authors.

Flat Sheet Membrane Test Module	Pressure (bar)	Temperature (°C)	PWF (L/m^2^h)	References
Plate frame module (DSS LabStak M20, Alfa Laval Nakskov, Denmark) (crossflow)	8	-	14	Galanakis et al. (2011) [38]
Dead-end, cross-rotational (CR) 250-filter	3	70	84	Sainio et al. (2013) [39]
Crossflow plate-and-frame module (DSS LabStak M20, Alfa Laval Nakskov, Denmark)	4.03	20	13.5	Varol et al. (2013) [40]
Plate-and-frame module (DSS LabStak, Alfa Laval Nakskov, Denmark)	5	20	15.4	Sun et al. (2015) [41]
Plate-and-frame module (DSS LabStak, Alfa Laval Nakskov, Denmark)	10	20	17.5	Sun et al. (2015) [41]
-	5.5	50	13.1	Li et al. (2019) [42]
Alfa Laval, Lab-Uni 20 Plate-and-frame, Nakskov, Denmark	4	25	4	Cabral et al. (2019) [43]
Alfa Laval, Lab-Uni 20 Plate-and-frame, Nakskov, Denmark	8	25	9.5	Cabral et al. (2019) [43]
Crossflow filtration system	9	25	58	Manios et al. (2023) [44]
Triple System Model F1 crossflow	5.6	20	20.16	This work
Triple System Model F1 crossflow	10.1	25	92.84	This work

**Table 4:** Comparison of permeability coefficients of native and modified membranes before and after filtering the pollutant.

Membrane	Aw ∙ 10^8^ (s/m) Before SMX Filtration	Aw ∙ 10^8^ (s/m) After SMX Filtration
Native	2.0881	-
MgCl_2_ (0.5 g/L) modified	-	0.4671
MgCl_2_ (1.0 g/L) modified	0.4671	0.7358
rGO modified	0.1257	0.0794
Chitosan modified	1.0885	0.4506

## Data Availability

The original contributions presented in the study are included in the article, further inquiries can be directed to the corresponding author.
